# Supplementary material for: Introgression shapes fruit color convergence in invasive Galápagos tomato
Source: eLife. 2021 Jun 24;10:e64165. doi: 10.7554/eLife.64165 (PMC8294854; doi:10.7554/eLife.64165)
Supplement: Transparent reporting form [file elife-64165-transrepform.pdf]

eLIFE
1st Floor
24 Hills Road
Cambridge CB2 1JP, UK
P 01223 855340
W elifesciences.org
T @elife

eLife's transparent reporting form

We encourage authors to provide detailed information *within their submission* to facilitate the interpretation and replication of experiments. Authors can upload supporting documentation to indicate the use of appropriate reporting guidelines for health-related research (see EQUATOR Network), life science research (see the BioSharing Information Resource), or the ARRIVE guidelines for reporting work involving animal research. Where applicable, authors should refer to any relevant reporting standards documents in this form.

If you have any questions, please consult our Journal Policies and/or contact us: editorial@elifesciences.org.

Sample-size estimation

- You should state whether an appropriate sample size was computed when the study was being designed
- You should state the statistical method of sample size computation and any required assumptions
- If no explicit power analysis was used, you should describe how you decided what sample (replicate) size (number) to use

Please outline where this information can be found within the submission (e.g., sections or figure legends), or explain why this information doesn't apply to your submission:

No traditional power analysis was conducted. Sample sizes on the archipelago were determined by the presence/absence of wild populations. Within populations, we made care to sample at least 20 chromosomes (10 individuals) where possible, to facilitate population genetic analyses. Sample sizes on the mainland (accessions) were chosen to capture the entire native range. See Results section "Sequencing and collections", Methods sections "Population sampling and genotyping", "Demographic inference", and "Inferring gene flow between contemporary island populations", and Supplementary Text Section S1.

Replicates

- You should report how often each experiment was performed
- You should include a definition of biological versus technical replication
- The data obtained should be provided and sufficient information should be provided to indicate the number of independent biological and/or technical replicates
- If you encountered any outliers, you should describe how these were handled
- Criteria for exclusion/inclusion of data should be clearly stated
- High-throughput sequence data should be uploaded before submission, with a private link for reviewers provided (these are available from both GEO and ArrayExpress)

Please outline where this information can be found within the submission (e.g., sections or figure legends), or explain why this information doesn't apply to your submission:

Not applicable. We did not perform any traditional experiments requiring replication.

eLife Sciences Publications, Ltd is a limited liability non-profit non-stock corporation incorporated in the State of Delaware, USA, with company number 5030732, and is registered in the UK with company number FC030576 and branch number BR015634 at the address 1st Floor, 24 Hills Road, Cambridge CB2 1JP | August 2014

Statistical reporting

- Statistical analysis methods should be described and justified
- Raw data should be presented in figures whenever informative to do so (typically when N per group is less than 10)
- For each experiment, you should identify the statistical tests used, exact values of N, definitions of center, methods of multiple test correction, and dispersion and precision measures (e.g., mean, median, SD, SEM, confidence intervals; and, for the major substantive results, a measure of effect size (e.g., Pearson's r, Cohen's d)
- Report exact p-values wherever possible alongside the summary statistics and 95% confidence intervals. These should be reported for all key questions and not only when the p-value is less than 0.05.

Please outline where this information can be found within the submission (e.g., sections or figure legends), or explain why this information doesn't apply to your submission:

We make extensive use of several existing bioinformatic, statistical, and population genetic methods in all sections of our methods/results and report all relevant summary statistics. See Methods sections "Phylogenetic reconstruction", "Demographic inference", "Inferring gene flow between contemporary island populations", Results sections "Genetic data support an Ecuadorian origin for most invasive populations", "Demographic reconstruction supports a recent colonization by PIM on the Galápagos", "Admixture analyses support the occurrence of inter- and intraspecific gene flow", "An introgressed origin for orange fruits in PIM", and Figure 2 legend, Table 2 legend, Figure 3 legend, Figure 4 legend, Table S14 legend, and Table S15 legend.

We also implemented a custom hidden Markov model to detect introgression and provide extensive documentation of its structure in Methods section "Introgression analysis", Results section "Admixture analyses support the occurrence of inter- and intraspecific gene flow", and Supplementary Text Section S5. Scripts for our HMM are also available through Dryad and GitHub repositories.

(For large datasets, or papers with a very large number of statistical tests, you may upload a single table file with tests, Ns, etc., with reference to sections in the manuscript.)

Group allocation

- Indicate how samples were allocated into experimental groups (in the case of clinical studies, please specify allocation to treatment method); if randomization was used, please also state if restricted randomization was applied
- Indicate if masking was used during group allocation, data collection and/or data analysis

Please outline where this information can be found within the submission (e.g., sections or figure legends), or explain why this information doesn't apply to your submission:

Groups assigned in our manuscript reflect species identity. We describe our taxonomic treatments in Supplementary Text Section S1.

Additional data files ("source data")

- We encourage you to upload relevant additional data files, such as numerical data that are represented as a graph in a figure, or as a summary table
- Where provided, these should be in the most useful format, and they can be uploaded as "Source data" files linked to a main figure or table
- Include model definition files including the full list of parameters used
- Include code used for data analysis (e.g., R, MatLab)
- Avoid stating that data files are "available upon request"

Please indicate the figures or tables for which source data files have been provided:

All relevant data and code have been deposited in two repositories: Dryad (https://datadryad.org/stash/share/zZqtThkaRnSrQNrVV0hXzbALSK4XoZKrYYVJRPq0vq4) and GitHub (https://github.com/gibsonMatt/galtom). This is in addition to raw sequence data available through NCBI SRA (BioProject PRJNA661300).
